# Patient and rescuer safety: recommendations for dispatch and prioritization of rescue resources based on a retrospective study of Norwegian avalanche incidents 1996–2017

**DOI:** 10.1186/s13049-019-0585-7

**Published:** 2019-01-14

**Authors:** Albert Lunde, Christen Tellefsen

**Affiliations:** 1 University of Stavanger, 4036 Stavanger, Norway; 2 The Norwegian Air Ambulance, Dombås, Norway

**Keywords:** Snow avalanche, Accidents, Rescue operations, Patient safety, Rescuer safety, Risk management, Dispatch, Rescue resources

## Abstract

**Background:**

Avalanche emergency response should address current accident scenarios to optimize survival chances of victims and to keep rescuers safe. The purpose of this article is to present a basis for evaluation and necessary adjustments in dispatch, prioritization, and management of Norwegian avalanche rescue operations.

**Methods:**

This is the first peer-reviewed retrospective study of all Norwegian avalanche incidents registered by the two Joint Rescue Co-ordination Centers (JRCCs) in the period 1996–2017 that describes the characteristics and trends of rescue missions and victims.

**Results:**

The Norwegian JRCCs have registered 720 snow avalanche events, with a total of 568 avalanche victims, of which 120 (21%) died. Including those fatally injured, a total of 313 avalanche victims in 209 accidents were treated as patients (55%), and we saw > 1 patient in 24% of these operations. Norwegian avalanche victims were partially or completely recovered prior to the arrival of rescuers in 75% (*n* = 117) of all rescue operations. In the remaining 25% of cases, the rescue service located 62% (*n* = 55) of the avalanche victims visually or electronically. In 50% of the 720 incidents, rescuers spent time searching in avalanches with no victims.

**Conclusions:**

This survey indicates that we have experienced a shift in Norwegian avalanche rescue: from search for missing persons in the avalanche debris to immediate medical care of already-located patients. The findings suggest that a stronger focus on both patient and rescuer safety is necessary. The patients must be ensured the right treatment at the right place at the right time and the allocation of rescue resources must reflect a need to reduce exposure in avalanche terrain, especially in cases with no affirmed victims. We present a flowchart with a recommended rescue response to avalanche accidents in Norway.

## Background

Norway’s National Guidelines for Avalanche Rescue [1] give directions for the management of avalanche rescue operations. The main aim is safe and efficient rescue efforts and the rescue service work to ensure that avalanche victims receive as early and advanced emergency medical treatment as other patients in the country [2, 3]. The existing guidelines state that rescue dogs and volunteer rescue specialists be summoned and dispatched along with the first responding National Air Ambulance Service (NAAS) [4]. This routine normally causes a delay in air rescue response because the specialized resources are not stationed at the helicopter base.

It is important to customize the avalanche rescue response to the avalanche victims’ need for medical assistance, the capacity of the air rescue services [5], and the safety of rescuers. Mair et al. [6] concluded that: “Medical emergencies are encountered at avalanche scenes twice as often as there is need to search for totally buried victims, clearly supporting the immediate dispatch of medical crewmembers to the accident site”. The important parameters are the patients’ expected survival time and the rescue time (time from the accident until treatment is initiated), given by the patients’ injuries and the emergency preparedness of the rescue service. Although asphyxia is the main cause of death in avalanche victims, several studies underline that many victims die because of mechanical injuries [7–9], especially to the chest and head. This is also the case for patients who are not totally buried [7, 10, 11]. Hermann Brügger directs attention to a lack of consideration of trauma in the field management algorithm for the care of avalanche victims [12]; this should be reflected also in dispatch routines. Avalanche emergency preparedness has improved with standardized avalanche rescue training for all Norwegian air rescue crews [5] and the provision of electronic search devices (transceivers and RECCO) to all air ambulance and rescue helicopter bases. Avalanche rescue methodology, which was formerly a domain of the volunteer rescue organizations, is now a part of standard operating procedures for all actors in the rescue service [1] and Helicopter Emergency Medical Service.

In this study, we present a basis for evaluating dispatch and prioritization of avalanche rescue resources. The data describe important characteristics of rescue operations, victims, location methods, and the situation at the accident site when rescuers arrive.

## Methodology

### Data selection

To evaluate Norwegian avalanche rescue operations, the JRCCs authorized the first author to collect and organize data from rescue logs and reports since 1996. The National Police Directorate has granted access to police rescue logs for the same period. Eighty-three avalanche rescue variables were extracted from operational data and text fields, anonymized, and recorded in a Microsoft Excel database, hereafter named the Norwegian Avalanche Rescue Database. The variables describe time and place, incident type, avalanche size, victims, rescue resources, rescue response time, location methods, weather, regional avalanche danger level, and risk level [13, 14].

**Fig. 1:**
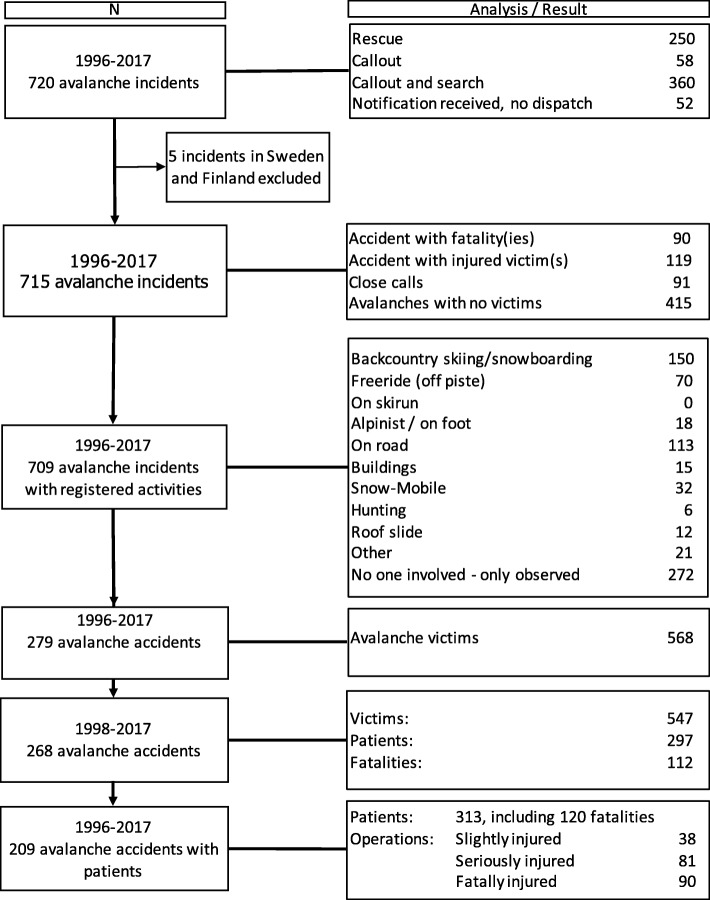
Norwegian avalanche incidents 1996–2017. Flowchart showing selections of Norwegian avalanche incidents and key results, based on logs from the Norwegian Joint Rescue Coordination Centers for northern and southern Norway 1996–2017

The incidents comprise all outdoor activity categories and infrastructure-related avalanches. We included all or a selection of the incidents in the study, based on type, amount, and quality of information linked to each incident (Fig. 1), thus, the number of incidents and observations will vary between analyses. As appropriate, five Swedish and Finnish incidents that were assisted by the Norwegian Rescue Service, were excluded in all analyses concerning characteristics of avalanches, victims, and patients. We concentrated specific analyses on accidents with confirmed victims in the two 10-year periods from 1998 to 2017 (*n* = 268). When describing the situation at the accident site when the rescuers arrived, we selected all cases with available relevant information in the time period 2010–2017 (*n* = 117).

### Definitions

An avalanche incident is any recorded event, with or without confirmed avalanche victims. An accident is an event with people caught by an avalanche, with three categories: fatal accident, personal injury accident, and close call. Vehicles and houses involved in avalanches, without passengers or inhabitants directly affected by debris, were counted as close calls. Persons directly affected by the avalanche debris were counted as victims. All victims with any degree of physical injury were defined as patients. Data about sensitive and detailed information about the patients’ conditions has not been recorded. Hence, degree of injury was defined in accordance with common practice in road traffic accidents; *“slight injury”* (treated on site or at a local doctor’s office), *“serious injury”* (evacuated to a hospital for examination and treatment) and *“fatal injury”*. Fatalities were recorded without reference to length of hospitalization following the accident.

### Statistical analysis

We used Microsoft Excel Data analysis with XLSTAT [15] for statistical analyses. To characterize rescue operations, avalanche victims, patients and fatalities, we calculated frequencies, rates, mean, median, interquartile range, range and 95% confidence interval (indicated by ±). Indications of monotonic trends over time were analyzed using the Mann-Kendall trend test [16, 17] and the Kolmogorov-Smirnov test [18] was applied to detect similarities of distributions. The two-Sample t-test was used to compare group means when comparing two different time periods. We considered bilateral *p* values below 0.05 significant.

## Results

### Avalanche rescue operations, victims, patients and fatalities

In the period 1996–2017, the JRCCs in Norway registered a total of 720 snow avalanche incidents, with an annual mean of 33 ± 7 (Fig. 1). Apart from non-involvements (38%, *n* = 709), the most frequent avalanche incidents were related to backcountry skiing (21%) and roads (16%). In 360 of the 720 incidents, rescuers were called out and searched in avalanches without any involved victims. A rescue response in avalanche accidents was the case in 35% of all incidents. In the remaining 15% of incidents, rescuers were dispatched, but not activated on site.

There were 568 avalanche victims in 279 accidents. Of the recorded incidents, 58% (*n* = 715) had no victims and 13% were close calls, leaving 209 (29%) personal injury and fatal accidents. These accidents comprised 313 patients, including 120 fatalities.

**Fig. 2:**
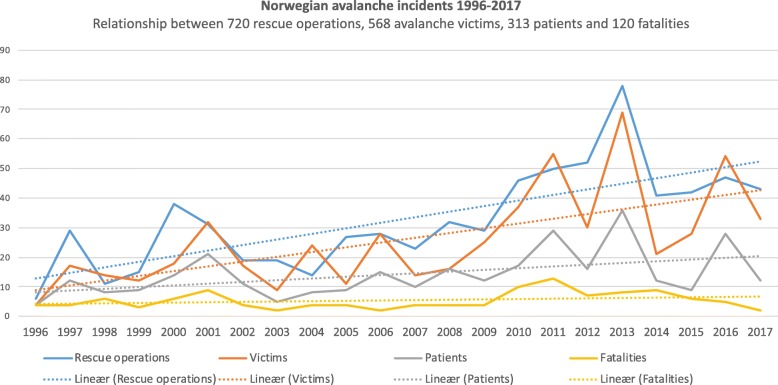
Norwegian avalanche rescue statistics 1996–2017. Relationships among 720 Norwegian avalanche rescue operations, 568 avalanche victims, 313 patients and 120 fatalities in the 22-year-period from 1996 to 2017, based on JRCC information

Figure 2 illustrates the time series 1996–2017, based on the number of rescue operations, avalanche victims, registered patients, and fatalities. The distributions of victims vs. patients and patients vs. fatalities were significantly different, with *p* values 0.017 and < 0.0001, respectively. Comparing victims and patients in 1996–2007 and 2008–2017, only the last time period showed significantly different distributions (*p* value: 0.041).

We found statistically significant increases in the number of rescue operations, victims, and patients, as all these parameters nearly doubled in the period 2008–2017 compared with 1998–2007. No significant difference was found between the mean numbers of avalanche fatalities in the two time periods 1998–2007 and 2008–2017, although the annual average increased from 4.4 to 6.9.

The period from 1998 to 2017 showed 0.55 patients per avalanche victim. The trend tests showed a slight, though significant, decrease in the ratio of patients to victims (*p* value = 0.009, Sen’s slope: − 0.013). We also noted a significant decrease in the number of fatalities per victim (*p* value = 0.027, Sen’s slope: − 0.01). There was no trend in the series of fatalities per patients, with a ratio of 0.38.

### Avalanche fatalities

The number of fatalities in avalanche accidents showed an average of 5.5 deaths per year over the period 1996–2017. The overall mortality rate of avalanche victims in the same period was 0.21 (*n* = 568). From 2010, we observed a marked change from the preceding 14-year annual average of 4.3 avalanche-related deaths. From 2010 through 2017, the annual average nearly doubled, to 7.5 fatalities per year (*p* value = 0.032). The number of fatalities fluctuated over shorter time periods: the number of deaths declined from an annual average of 8.0 fatalities from 2012 through 2014 to 4.3 from 2015 through 2017 (*p* value = 0.071).

Specific mention should be made regarding people falling off breaking cornices, as they are counted as avalanche accidents in Norway. This event caused 11 fatalities and 3 seriously injured patients in the 22-year period from 1996 to 2017. Eleven (two survivors) were located by organized rescuers and only three by companions. Nine were found because of visible parts, three by transceivers, and two by probing. Six of these victims were foreign citizens.

### What is the situation at the accident site on arrival of rescuers?

#### Multiple victims and patients

The most frequent scenarios of the 209 rescue operations with patients to assist during 1996–2017 were seriously injured patients (81) and fatalities (90), with fewer (38) for slightly injured people. The average number of patients per rescue operation was 1.5 (range: 7), with > 1 patient in 24% of operations. There was a small but not significant (nsd) increase in the frequency of rescue operations with ≥3 avalanche victims to assist, from 17.9% of all operations during 1998–2007 (*n* = 95) to 21.3% during 2008–2017 (*n* = 174). Likewise, for ≥3 patients, from 6.3 to 10.3% (nsd) of all rescue operations.

### Situation on arrival of rescuers

Overall, during 1996–2017, NAAS helicopters and Air-Force-operated search-and-rescue helicopters (330 Squadron) responded to 325 and 184 of the 720 registered avalanche incidents, respectively. NAAS personnel were the most frequent first organized rescue responders (26.8%, *n* = 440). They brought rescue dogs or avalanche rescue specialists to 37 and 24 of these 118 rescues, respectively. The median response time (time from the emergency call to first arrival) was 40 min (IQR: 30.5, *n* = 191). The rescue time (time from accident until the patient is offered first aid) may be much longer because of difficult access, search, or extrication. In this dataset, rescue time was only recorded in 62 accidents, with a median time of 85 min (IQR: 123.8).

Table 1 illustrates that the most common situation facing first responding rescuers in the period 2010–2017 (*n* = 117) was already-localized and more or less extricated (75.2%) and injured (82.9%) victims.

**Table 1:** Norwegian avalanche accidents 2010–2017

	Fatal accident	Injured victims	Close call	Total	%
Not localized	22	6	1	29	24.8
Localized-not excavated	1	3	2	6	5.1
Localized and excavated	16	49	17	82	70.1
Total	39	58	20	117	
%	33.3	49.6	17.1		

Situation at the scene of the accident on arrival of rescuers, relative to type of accident. *N* = 117

In 29 accidents, 55 victims still had to be located by arriving rescuers. Thirty-nine of these victims died (70.9%). Thirty-four (62%) were located without specialized search resources (dogs and divers) or increased manpower for efficient probing and digging (Table 2). The high number of victims located by digging was caused by avalanches hitting buildings in Svalbard in 2015.

**Table 2:** Rescuers’ methods of locating 55 victims in 29 Norwegian avalanche accidents of all categories in the period 2010–2017

Location method	Visible	Probing	Dog	Transceiver	Diving	Audible	Digging
N	17	5	6	14	1	2	9
%	30.9	9.1	10.9	25.5	1.8	3.6	16.4

### Avalanche victims and their rescuers

Sufficient information allowed categorization of rescue strategies (self-rescue, companion rescue and organized rescue) for 416 of the 568 avalanche victims in the period 1996–2017. These categories include all kinds of activities and rescue situations, irrespective of degree of burial and need of assistance. The largest proportion comprised those who ended up on the surface or who managed to free themselves from the avalanche debris (35.6%, *n* = 148). In companion rescue situations (29.3%, *n* = 122), 86.1% of the victims survived, whereas the proportion of survivors who had to wait for organized rescuers (35.1%, *n* = 146) was 41.1%.

### Methods of locating avalanche victims

**Fig. 3:**
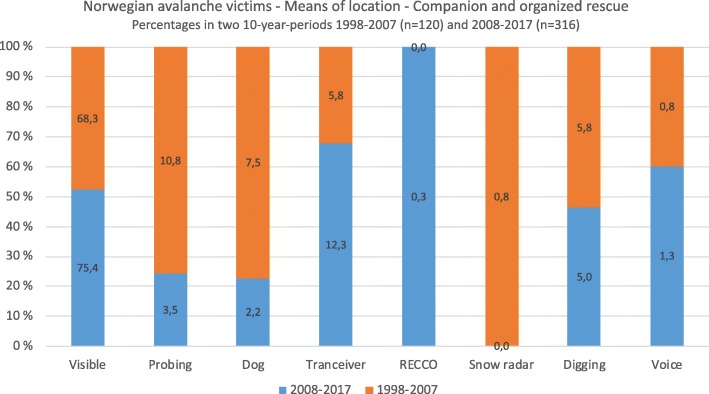
Methods of locating avalanche victims in Norway over two time periods; 1998–2007 and 2008–2017

Between 2008 and 2017 (*n* = 316), 89% of victims (visible avalanche victims [75.4%], those located by transceivers [12.3%], and those who were able to call for help [1.3%]) did not require specialized search resources to be located (Fig. 3). In practice, probing and search dogs are equally successful (in 3.5 and 2.2% of cases, respectively), though probing by companions is also included in this dataset.

The proportion of avalanche victims found by visual searching increased slightly between 2008 and 2017, from 68.3 to 75.4% (*p* value: 0.004). The major difference between the periods 1998–2007 and 2008–2017, however, is the increase in the number of avalanche victims who were located using transceivers, from 5.8 to 12.3% (*p* value < 0.001).

## Discussion

The observed increase in the number of rescue operations, victims, and patients places an extra burden on both health and rescue resources. The high number of rescue operations with no victims requires specific attention because rescuers already spend ample time in avalanche runout zones in real rescue situations [14, 19]. Mair et al. [6] recommend a limited response in cases where witnesses cannot give clear information about victim involvement. We should develop better systems to handle uncertainty in the first phase of avalanche rescue operations.

In this study, every second avalanche victim was a patient, which approximates the findings of Hohlrieder et al. [20], which showed 49 injuries in 105 victims. Avalanche victims are exposed to considerable mechanical pressure [21] and forceful impacts with terrain features. The entire range of mechanical injuries is possible [22] and victims may sustain permanent impairment. In Canada, “trauma accounted for more than half of the deaths among people extricated in the first 10 minutes” [23] and other studies also direct attention to injured avalanche victims [6, 8, 24]. Survivors are frequently exposed to low temperatures, wind, and moisture, which all contribute to lower core temperature and an increased tendency to bleeding [25]. Therefore, early and proficient medical treatment of avalanche victims is important.

It is worrying that multiple victim scenarios are so common. In a recent study by Kottmann et al. [26], 32% of all rescue operations had more than one casualty. This phenomenon should be addressed in both emergency response and accident prevention.

The downward trends in numbers of patients and fatalities per victim may reflect an increase in successful rescues, which results from both swift victim location, use of safety equipment, and successful prehospital patient care. The ratio of patients per avalanche victim indicates victim vulnerability. Equipment like transceivers, probes and shovels contributes to reduced burial time, and backpacks with floatation devices may prevent victims from being totally covered by debris [27, 28]. However, none of these offer any direct or extensive protection. Safety helmets are recommended for recreational skiers and snowboarders [29] and “should be considered when travelling in avalanche terrain” [22]. The important message for dispatch centers is that avalanche victims wearing safety equipment will likely be found and excavated before the arrival of rescuers, and they are probably injured.

The varying ratio between avalanche victims and the number of fatalities emphasizes that coincidence rules the outcome of these accidents. Those who end up totally covered by avalanche debris are dependent on rescue-competent companions to increase their likelihood of survival. This study indicates that mountain travelers are increasingly aware of their rescue responsibility because more avalanche victims have been located visually and by using transceivers. Consequently, it is unusual for the Norwegian rescue service to have to search for missing people in avalanches. If so, the victims are often visible or searchable with transceivers or RECCO.

**Fig. 4:**
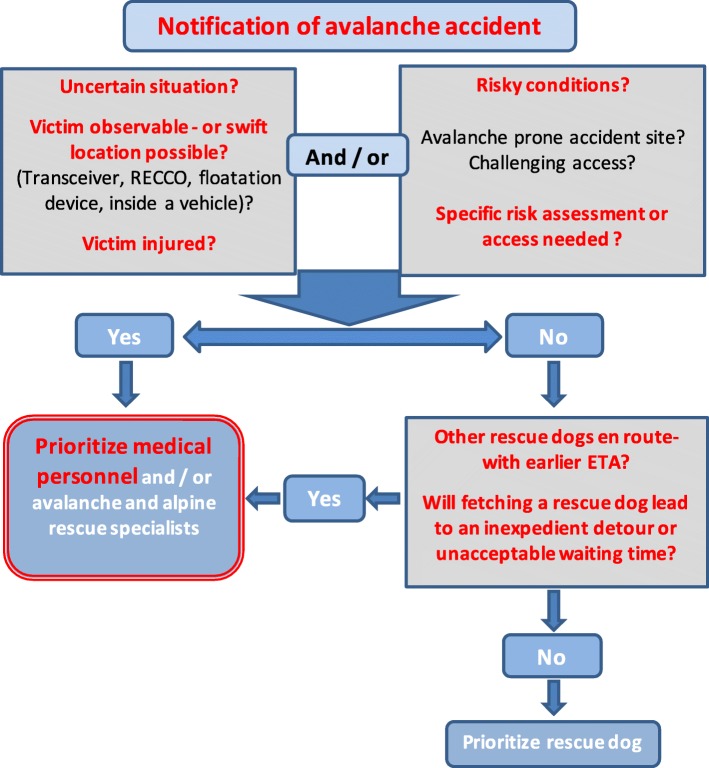
Flowchart showing the recommended rescue response to avalanche accidents in Norway

The length of burial time and burial depth affect survival rate [7]. This connection has rightly guided development of emergency preparedness and rescue response for years. The parallel introduction of more efficient means of companion rescue has caused a change in the initial tasks of the first responding organized rescuers [30]. We should adjust the allocation of rescue resources to reflect current rescue scenarios and ensure that patients get the right treatment at the right place at the right time [31]. Avalanche accidents must be perceived and handled as acute medical emergencies. This is reflected in today’s emergency preparedness system, as air ambulance helicopters are frequent first organized rescue responders. However, if we aim to save the most critically-injured patients, prehospital medical personnel should respond without first waiting or detouring for voluntary search resources. In most cases, teaming up with extra medical personnel may be more pertinent than fetching manpower and dogs for searching. The findings of this study, and the conclusions of Mair et al. [6], provide the basis for a flowchart (Fig. 4) for dispatching and prioritizing resources in avalanche rescue operations in Norway.

### Limitations

Our Excel database was developed in retrospect and some of the information has been interpreted from free text fields. Even if the informational quality of registration has increased over the years, it still varies because of both inter-operator differences in registration of relevant details and inter-regional differences in conducting and documenting rescue activities. To compensate for a lack of information in logs and reports from relevant authorities, we have also included reports from the mass media. In some cases, both rescuers and involved victims have been asked to supply relevant details. Nevertheless, some parameters suffer from varied levels of registration detail, which causes a bias in evaluation of time series and trends, especially in cases without fatalities or injured persons.

## Conclusion

We have experienced a shift in Norwegian avalanche rescue from searching for missing persons in the avalanche debris to providing immediate medical care of already-located patients. Subsequently, the rescue service’s longstanding focus on rapid implementation of effective search operations should be changed to speedy and safe provision of advanced prehospital emergency medical treatment and evacuation.

Dispatch routines should reflect that many rescue operations take place in adverse conditions that threaten rescuer safety. Especially in situations of uncertainty about victim involvement, necessary actions to remain in control of the situation should be taken on all managerial levels, even if this means a slower and more limited rescue response.

## Abbreviations

IQR: Interquartile range
JRCC: Joint Rescue Coordination Centers in South and North Norway
NAAS: National Air Ambulance Service
RECCO: A two-part system comprising a detector used by organized rescue groups and reflectors that are integrated in mountain travelers’ clothes or gear
